# Prevalence of Shift Work Disorder: A Systematic Review and Meta-Analysis

**DOI:** 10.3389/fpsyg.2021.638252

**Published:** 2021-03-23

**Authors:** Ståle Pallesen, Bjørn Bjorvatn, Siri Waage, Anette Harris, Dominic Sagoe

**Affiliations:** ^1^Department of Psychosocial Science, University of Bergen, Bergen, Norway; ^2^Norwegian Competence Center for Sleep Disorders, Haukeland University Hospital, Bergen, Norway; ^3^Optentia, The Vaal Triangle Campus of the North-West University, Vanderbijlpark, South Africa; ^4^Department of Global Public Health and Primary Care, University of Bergen, Bergen, Norway

**Keywords:** review, meta-regression, meta-analysis, prevalence, shift work disorder, shift work sleep disorder

## Abstract

**Objectives:** No systematic review or meta-analysis concerning the prevalence of shift work disorder (SWD) has been conducted so far. The aim was thus to review prevalence studies of SWD, to calculate an overall prevalence by a random effects meta-analysis approach and investigate correlates of SWD prevalence using a random-effects meta-regression.

**Methods:** Systematic searches were conducted in ISI Web of Science, PsycNET, PubMed, and Google Scholar using the search terms “shift work disorder” and “shift work sleep disorder.” No restrictions in terms of time frame were used. Included studies had to present original data on the prevalence of SWD in an occupational sample published in English. A total of 349 unique hits were made. In all, 29 studies were finally included from which two authors independently extracted data using predefined data fields. The meta-regression included four predictors (diagnostic criteria, study country, type of workers, and sample size).

**Results:** The overall prevalence of SWD was 26.5% (95% confidence interval = 21.0–32.8). Cochran *Q* was 1,845.4 (*df* = 28, *p* < 0.001), and the *I*^2^ was 98.5%, indicating very high heterogeneity across the observed prevalence estimates. Diagnostic criteria (International Classification of Sleep Disorders-2 = 0, International Classification of Sleep Disorders-3 = 1) and sample size were inversely related to SWD prevalence.

**Conclusions:** The prevalence of SWD was high across the included studies. The between-study disparity was large and was partly explained by diagnostic criteria and sample size. In order to facilitate comparative research on SWD, there is a need for validation and standardization of assessment methodology as well as agreement in terms of sample restrictions.

## Introduction

Shift work implies working in different shifts, e.g., morning, evening, and night, and can vary along several dimension, such as intensity and speed of rotating (European Parliament of the Council, 2003). Night work, which is a special type of shift work, can be defined as work that covers at least 3 h of work between 11 p.m and 6 a.m (Garde et al., 2019). Still, it should be noted that different studies use definitions that may deviate somewhat from the aforementioned ones. Shift work and night work are common work schedules, and 2017 data from the European Union show that these include 21 and 19% of the workforce, respectively (Eurofound, 2017).

A vast amount of studies show that shift and night works negatively impact health. Such working arrangements, for example, have been associated with cardiovascular disease (Torquati et al., 2018), cancers (Wang et al., 2015; Gan et al., 2018; Pahwa et al., 2018), metabolic disturbances (Watanabe et al., 2018; Gao et al., 2020), sleep disturbances (Pallesen et al., 2010), gastrointestinal disorders (Knutsson and Bøggild, 2010), and impaired reproductive health (Stocker et al., 2014), as well as impaired mental health (Torquati et al., 2019). Furthermore, shift work and night work have also been linked to negative organizational outcomes such as accidents (Fischer et al., 2017), impaired cognitive efficiency (Di Muzio et al., 2020), sick leave (Merkus et al., 2012), low job satisfaction (Jamal, 1981), and turnover and turnover intention (Pisarski et al., 2006; Flinkman et al., 2008). The underlying mechanisms for the negative health consequences are not fully understood but involve most likely circadian disruption leading to neuroendocrine and cardiometabolic stress, curtailed and disturbed sleep causing altered immune functioning and cellular stress, and risk behaviors and psychosocial stress with cognitive impairment and poor emotion regulation as consequences (Kecklund and Axelsson, 2016).

When a shift or night worker experiences sleep disturbance that is associated with clinically significant distress or impairment of social, occupational, or other areas of functioning, he/she may be suffering from shift work disorder (SWD; Wright et al., 2013). SWD was termed “shift work sleep disorder” in the first edition of the International Classification of Sleep Disorders (American Sleep Disorders Association, 1990). According to the second edition of the International Classification of Sleep Disorders (ICSD-2), SWD is diagnosed on four essential points: (1) a complaint of insomnia or excessive sleepiness that is temporally associated with a recurring work schedule that overlaps the usual time for sleep; (2) the symptoms are associated with the shift work schedule over the course of 1 month; (3) sleep log or actigraphy monitoring for at least 7 days demonstrates disturbed circadian and sleep-time misalignment; and (4) the sleep disturbance is not better explained by another current sleep disorder, medical or neurological disorder, mental disorder, medication use, or substance use disorder (American Academy of Sleep Medicine, 2005). When the third edition of the diagnostic system (ICSD-3) was released in 2014, three notable amendments of the diagnostic criteria for SWD were made: (1) the insomnia/sleepiness complaint must be accompanied by a reduction of total sleep time; (2) the duration of the symptoms must be at least 3 months; and (3) sleep log or actigraphy monitoring has to be conducted for at least 14 days and needs to include both work and free days (American Academy of Sleep Medicine, 2014).

As insomnia is the most prevalent sleep disorder, a plausible means of evaluating whether SWD is highly prevalent in the shift-working population is by comparing SWD prevalence to the prevalence of insomnia among daytime workers. In this regard, insomnia prevalence among daytime workers varies, but has been estimated at 27.6% in home nursing caregivers in Japan (Takahashi et al., 2008), 8.5% (Yong et al., 2017) and 18.0% (Drake et al., 2004) in two large US cross-occupational samples, 9.9% in a Norwegian cross-occupational sample (Ursin et al., 2009), and 12.4% in textile factory workers in Iran (Yazdi et al., 2014).

Although sleep diaries or actigraphic recordings are requirements for a formal diagnosis, most large-scale epidemiological studies have estimated SWD prevalence based on different types of self-report questionnaires. Across these, no consensus in terms of how to estimate SWD prevalence seems to have been established. Some emphasize the differential prevalence of the core symptoms (insomnia or sleepiness) between day workers and shift/night workers to make proper estimates (Drake et al., 2004). Others assess SWD with a specific questionnaire based on a discrimination function analysis (Barger et al., 2012), whereas other scholars anchor their estimates in a minimum of questions adhering as closely as possible to formal diagnostic criteria (Waage et al., 2009). Another unresolved matter concerns who can be diagnosed. Some reserve this for night workers only (Rajaratnam et al., 2011), whereas others argue that SWD may even affect day workers (Flo et al., 2012), as it is conceivable that night owls, for example, having day work with an early start, will work at a time overlapping with their usual sleep time (Facer-Childs et al., 2019). These factors may impact the estimated prevalences of SWD. Another factor that should be taken into consideration is that of work hours. Developing countries typically exhibit longer work hours on average, frequently >40 h per week, compared to developed countries where work hours are concentrated in the range of 30–40 h per week (Messenger and Ray, 2013). As long work hours may interfere with sleep (Virtanen et al., 2009) and cause excessive sleepiness (Wilsmore et al., 2013), study country may moderate prevalence estimates of SWD. Moreover, although sample size (e.g., small study effect) probably affects estimates less often in prevalence studies than in trial studies, sample size can nevertheless not be discounted as a moderator (Richter et al., 2019).

So far, neither a systematic review nor meta-analysis regarding the prevalence of SWD has been conducted. Against this backdrop, we aimed at: (1) presenting an overview of the current published literature on the prevalence of SWD, (2) synthesizing the prevalences using a random effects meta-analysis, and (3) exploring the correlates of potential significant between-study heterogeneity of SWD prevalence.

## Materials and Methods

### Search Strategy and Inclusion Criteria

We conducted a systematic and comprehensive literature search in Google Scholar, ISI Web of Science, PsycNET, and PubMed. The following keywords were used: “shift work disorder” OR “shift work sleep disorder.” A total of 645 hits (including the first 200 of 5,860 hits in Google Scholar) were identified from the database search. The Google Scholar search was conducted in order to identify gray literature. One record was identified through *ad hoc* searches. After removing duplicates, 349 records were available for screening. Of this pool, 41 records were removed after screening their titles. Next, the abstracts of the remaining 308 records were inspected. A total of 53 records were available after going through the abstracts. After screening the 53 full-text records for eligibility, 29 were included in the analysis.

**Figure 1:**
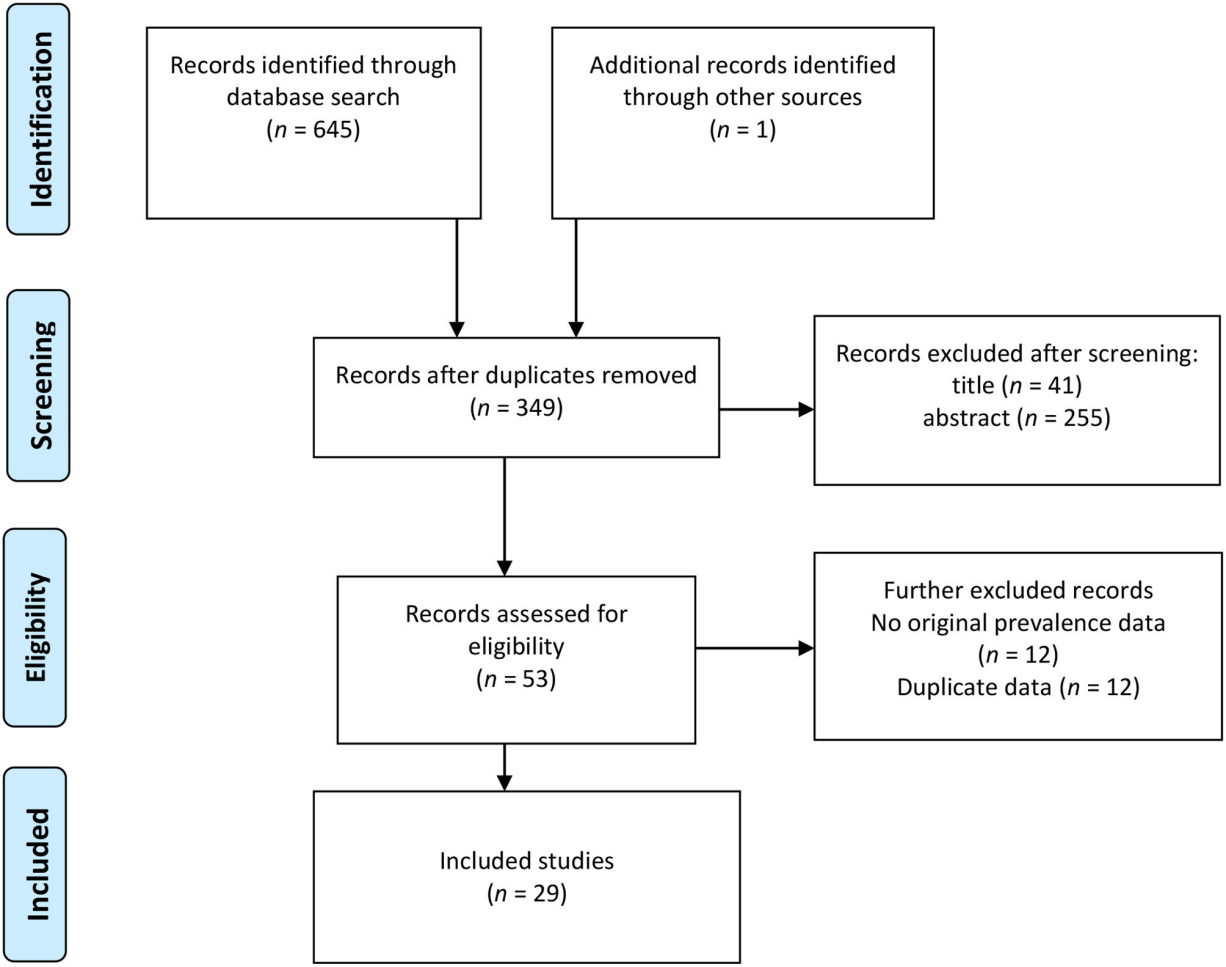
Flow diagram of systematic literature search on SWD prevalence.

The key inclusion criteria were that the study or record presented original data on the prevalence of SWD in an occupational sample and published in English. The literature search was conducted from February 12, 2020, to March 13, 2020. We conducted the literature search and selection in line with the Preferred Reporting Items for Systematic Reviews and Meta-Analyses (PRISMA) procedure (Moher et al., 2009), and the guidelines of the Meta-analysis of Observational Studies in Epidemiology (Stroup et al., 2000) group. Figure 1 presents the literature search and selection process. See Appendix for a completed PRISMA-guideline checklist.

### Data Extraction

The first and last authors independently conducted literature searches and selection of articles based on the aforementioned criteria. Using a standardized data extraction form, the following data were extracted from the identified studies and coded: first author name and publication year, data collection period, country, sample of shift workers, shift type, SWD assessment/measure, sample size (total, female, and male), age of the participants (range, mean ± *SD*), SWD prevalence, and response rate (Table 1). Discrepant extractions were resolved through discussion and further review until consensus was reached.

**Table 1:** Characteristics of prevalence studies of shift work disorder.

**References**	**Country**	**Shift sample**	**Shift type**	**SWD A**	**Night work**	***n***	***n* (Female)**	***n* (Male)**	**Age range**	**Age *M* ±*SD***	**T Prev %**	**RR %**
Anbazhagan et al. (2016)	India	Nurses	≥1 year experience	ICSD-2	Yes	130	130		Most 30–40	27.4 ± 2.6	43.1	
Asaoka et al. (2013)	Japan	Nurses	2 and 3 shifts	ICSD-2	No	993	993			30.0 ± 8.0	24.4	80.5
Barger et al. (2015)	United States	Firefighters	At least 1 night past month	ICSD-2	Yes	5,771					9.1	58.6
Booker et al. (2020)	Australia	Nurses	Rotating or permanent work, ≥15 h per week	ICSD-2	Yes	202	192	10	21–65	35.3 ± 12.0	29.2	42.5
Chen et al. (2020)	China	Nurses	2, 3, and random	ICSD-3	No	637	637		16–24	17.8 ± 1.5	37.7	95.2
Di Milia et al. (2013)	Australia	Community	All, no required position	ICSD-2	No	1,163	623	540	18–75^‡^	45.3 ± 11.2	15.1	50.0
Drake et al. (2004)	United States	Community	Rotating and nights past 2 weeks	ICSD-2	Yes	449					10.0	70.1
Fadeyi et al. (2018)	Nigeria	Nurses	Rotating	ICSD-2	Yes	44	32	12	26–45		43.2	96.7
Flo et al. (2012)	Norway	Nurses	All, ≥50% position	ICSD-2	No	1,968	1,773^‡^	187^‡^	21-63	33.1 ± 8.1^‡^	37.6	38.1
Francy et al. (2019)	India	Nurses	3 shifts	ICSD-2	Yes	120	120		22–42	27.1 ± 3.9	15.8	80.0^‡^
Haile et al. (2019)	Ethiopia	Nurses	2 and 3 shifts	ICSD-2	Yes	399	217	182	20–58	27.5 ± 5.6	25.6	94.3
Inoue et al. (2000)	Japan	Industry	Not specified	ICSD-1	Yes	332	18	314		37.9 ± 10.2	33.1	86.1
Joseph et al. (2018)	India	Nurses	3 shifts	ICSD-3	Yes	60			20–35 (91.6%)		0.0	
Kalmbach et al. (2015)	United States	Community	Rotating	ICSD-3	Yes	96	60			47.9 ± 13.3	18.8	
Kerkhof (2018)	The Netherlands	Community	Rotating, permanent early morning, permanent night	ICSD-3	No	250			21–60		12.4	
Kim et al. (2006)	South Korea	Nurses	Rotating	ICSD-2	Yes	87					32.2	
Lahtinen et al. (2019)	Finland	Airline	Early morning or night and evenings	ICSD-3	No	26		26		44.9 ± 9.0	65.4	100.0^‡^
Li and Fan (2018)	China	Industry	Not specified	ICSD-2	Yes	335					31.3	
Mizuno et al. (2016)	Japan	Flight controllers	≥3 nights work per month	ICSD-2	Yes	30	1				46.7	71.2
Rajaratnam et al. (2011)	United States, Canada	Police	≥1 night past month	ICSD-2	Yes	1,861					14.5	
Shi and Fan (2018)	China	Nurses	Not specified	ICSD-2	Yes	664				17.8 ± 1.6	35.8	
Stamatiou et al. (2016)	Greece	Health	Rotating, 32 h	ICSD-2	No	58			27–61	36.6	36.2	70.4
Tangkumnerd (2018)	Thailand	Nurses	All	ICSD-2	No	205	193	12	22–55	32.5 ± 7.3	23.9	74.2
Taniyama et al. (2015)	Japan	Industry	Rotating	ICSD-2	Yes	363		363	20–62	42.0 ± 8.8	62.8	82.0
Vanttola et al. (2020)	Finland	Hospital	≥3 nights per month	ICSD-2, ICSD-3	No	4,814	4,381		19–67^‡^	42.8 ± 11.5	ICSD-2: 24.1; ICSD-3: 7.3	69.0
Voinescu (2018)	Romania	Various	All	ICSD-3	No	488	351	137	18–65	32.6 ± 11.4	2.3	40.6
Vora et al. (2019)	India	Doctors, nurses	Not specified	ICSD-3	No	216				31.8 ± 7.7	22.2	61.7
Waage et al. (2009)	Norway	Oil rig workers	Swing	ICSD-2	Yes	103	5	98	19–59	39.8 ± 10.2	23.3	78.8
Zaki et al. (2016)	Egypt	Nurses	3 shifts and rotating	ICSD-2	Yes	150	150		20–35		84.0	53.6^‡^

*ICSD, International Classification of Sleep Disorders; Prev, prevalence; RR, response rate; SWDA, shift work disorder assessment; Waage et al. (2009)*.

‡ *Personal communication; Night work, whether or not all in the sample had a work schedule including night work*.

### Statistical Analysis

We used a random-effects model in the prevalence meta-analysis, using the DerSimonian and Laird approach for estimating the between-study variance (DerSimonian and Laird, 1986). Prevalence estimates and their corresponding 95% confidence intervals (95% CIs) were calculated. The preference for a random-effects model is based on its propensity for higher external validity or generalizability of findings and recommendation when included studies are assumed to represent different populations of studies (Borenstein et al., 2009). For between-study heterogeneity, we conducted a random-effects meta-regression analysis to examine whether the following predictors explained the heterogeneity in SWD prevalence: (*a*) diagnostic criteria (ICSD-2 vs. ICSD-3), (*b*) country of study; developing (China, Egypt, Ethiopia, India, Nigeria, Thailand) vs. developed (Australia, Canada, Finland, Greece, Japan, the Netherlands, Norway, Romania, South Korea, United States) countries, (*c*) type of work (includes night work: no/yes), and (*d*) sample size. As a scatter plot suggested a curvilinear relationship between prevalence (logit) and sample size, the latter variable was transformed by its natural logarithm before entering it to the regression model.

Heterogeneity was assessed using Cochran *Q*. The *I*^2^ statistic was calculated and reflects the proportion of variation in observed effects that is due to variation in true effects (Borenstein et al., 2017). An *I*^2^ of 0% suggests no heterogeneity, 25% indicates low heterogeneity, 50% indicates moderate heterogeneity, and 75% indicates high heterogeneity, respectively (Higgins et al., 2003). We also calculated the 95% prediction interval, which represents the interval within which the effect size of a future study would fall given that the study was randomly selected from the same population as the studies included in the present meta-analysis (IntHout et al., 2016). Publication bias was investigated using Egger test, denoting a regression model where the standardized effect size comprises the dependent variable and the inverse of the standard error is the independent variable. An intercept significantly different from zero suggests bias (Egger et al., 1997). Also, the trim-and-fill procedure by Duval and Tweedie (2000) was used for investigation of publication bias. This procedure is based on the funnel plot, where effect sizes are depicted along the *x* axis and where the inverse of the variance (sample size) is represented on the *y* axis. This creates a funnel plot with the largest and most precise studies situated at the top of the funnel. In the absence of publication bias, the funnel plot is symmetrical. Publication bias often entails lack of small studies with small effects. The trim-and-fill procedure trims off asymmetric outlying studies and replaces them with studies around the center, whereupon an adjusted effect size and 95% CI are calculated.

Additionally, we assessed study quality or risk of presenting biased prevalence estimates using a quality assessment checklist for prevalence studies (Hoy et al., 2012). The checklist comprises items reflecting 10 characteristics of the included studies, each scored 0 (low risk of bias) or 1 (high risk of bias). High risk was indicated by each of the following items: (1) study target population is not representative of the national working population, (2) sampling frame is not a representation of the target population, (3) random selection is not used, (4) response rate is <75%, (5) data are collected from a proxy, (6) an acceptable case definition is not used, (7) the study instrument is not shown to have reliability or validity, (8) same mode of data collection is not used for all subjects, (9) the shortest prevalence period for the parameter is not appropriate, and (10) one or more of the numerator(s) or denominator(s) is inappropriate. Hence, the total score ranged from 0 to 10 and was categorized as follows: high quality/low risk (0 to 3), moderate quality/risk (4 to 6), and low quality/high risk (7–10) (Table 2). The meta-analysis and metaregression analysis were conducted using the Comprehensive Meta-Analysis 3.0 software (Biostat Inc., 2014). When calculating the prevalences, the software logit transforms the prevalences in order to carry out all of the statistical analyses, before they are back-transformed to the metric of the prevalences. The transformation is based on the formula *Logit* = Ln*(p/(1–p))*, where *p* is the prevalence rate, and Ln, the natural logarithm. The formula for transforming the sampling variance (*V*) is *V*(*Logit*) = *1/np* + *1/n(1–p)*. The back-transformation is based on the following formula: *p* = *e*^*Logit*^*/(e*^*Logit*^ + *1)*, with *e* being the base of the natural logarithm.

**Table 2:** Risk of bias/methodological quality (Hoy et al., 2012) of included studies.

**References**	**1. *N* representative-ness**	**2. *N* frame**	**3. Randomization**	**4. Non-response bias**	**5. Primary data**	**6. Operationali-zation**	**7. Instrument**	**8. Consistency**	**9. Period**	**10. Estimation**	**Total risk score**	**Risk category^**§**^**
Anbazhagan et al. (2016)	1	0	0	1	0	0	1	0	0	0	3	Low
Asaoka et al. (2013)	1	0	0	0	0	0	1	0	0	0	2	Low
Barger et al. (2015)	1	0	0	1	0	0	1	0	0	0	3	Low
Booker et al. (2020)	1	0	0	1	0	0	0	0	0	0	2	Low
Chen et al. (2020)	1	1	1	0	0	0	1	0	0	0	4	Moderate
Di Milia et al. (2013)	1	0	0	1	0	0	1	0	0	0	3	Low
Drake et al. (2004)	1	0	0	1	0	0	1	0	0	0	3	Low
Fadeyi et al. (2018)	1	0	0	0	0	0	0	0	1	0	2	Low
Flo et al. (2012)	0	0	0	1	0	0	1	0	0	0	2	Low
Francy et al. (2019)	1	0	0	0	0	0	0	0	0	0	1	Low
Haile et al. (2019)	1	0	0	0	0	0	1	0	0	0	2	Low
Inoue et al. (2000)	1	1	1	0	0	0	1	0	1	0	5	Moderate
Joseph et al. (2018)	1	1	1	1	0	0	1	0	0	0	5	Moderate
Kalmbach et al. (2015)	1	0	0	1	0	0	1	0	0	0	3	Low
Kerkhof (2018)	1	1	1	1	0	0	0	0	0	0	4	Moderate
Kim et al. (2006)	1	1	1	1	0	0	0	0	1	0	5	Moderate
Lahtinen et al. (2019)	1	0	0	0	0	0	1	0	0	0	2	Low
Li and Fan (2018)	1	1	1	1	0	0	0	0	1	0	5	Moderate
Mizuno et al. (2016)	0	0	0	1	0	0	1	0	0	0	2	Low
Rajaratnam et al. (2011)	1	1	1	1	0	0	1	0	0	0	5	Moderate
Shi and Fan (2018)	1	1	1	1	0	1	1	0	1	0	7	High
Stamatiou et al. (2016)	1	0	1	1	0	1	1	0	1	0	6	Moderate
Tangkumnerd (2018)	1	0	0	1	0	0	1	0	0	0	3	Low
Taniyama et al. (2015)	1	1	1	0	0	0	1	0	0	0	4	Low
Vanttola et al. (2020)	1	0	0	1	0	0	1	0	0	0	3	Low
Voinescu (2018)	1	1	1	1	0	0	1	0	0	0	5	Moderate
Vora et al. (2019)	1	1	1	1	0	0	1	0	0	0	5	Moderate
Waage et al. (2009)	1	0	0	0	0	0	1	0	0	0	2	Low
Zaki et al. (2016)	1	0	0	1	0	0	0	0	0	0	2	Low

*Item score: (0: low risk, 1: high risk)*.

§ *Total quality/risk score: [range (0–10): high quality/low risk (0–3), moderate quality/risk (4–6), poor quality/high risk (7–10)]*.

## Results

### Description of Studies

Of the 29 included studies, publication years ranged from 2000 (Inoue et al., 2000) to 2020 (Booker et al., 2020; Chen et al., 2020; Vanttola et al., 2020). Studies were conducted in India (*k* = 4: Anbazhagan et al., 2016; Joseph et al., 2018; Francy et al., 2019; Vora et al., 2019), Japan (*k* = 4: Inoue et al., 2000; Asaoka et al., 2013; Taniyama et al., 2015; Mizuno et al., 2016), China (*k* = 3: Li and Fan, 2018; Shi and Fan, 2018; Chen et al., 2020), United States (*k* = 3: Drake et al., 2004; Barger et al., 2015; Kalmbach et al., 2015), Australia (*k* = 2: Di Milia et al., 2013; Booker et al., 2020), Finland (k = 2: Lahtinen et al., 2019; Vanttola et al., 2020), Norway (*k* = 2: Waage et al., 2009; Flo et al., 2012), and one study each from the following countries: Canada/United States (Rajaratnam et al., 2011), Egypt (Zaki et al., 2016), Ethiopia (Haile et al., 2019), Greece (Stamatiou et al., 2016), the Netherlands (Kerkhof, 2018), Nigeria (Fadeyi et al., 2018), Romania (Voinescu, 2018), South Korea (Kim et al., 2006), and Thailand (Tangkumnerd, 2018).

Samples were predominantly nurses (*k* = 13: Kim et al., 2006; Flo et al., 2012; Asaoka et al., 2013; Anbazhagan et al., 2016; Vedaa et al., 2016; Zaki et al., 2016; Fadeyi et al., 2018; Joseph et al., 2018; Shi and Fan, 2018; Tangkumnerd, 2018; Francy et al., 2019; Haile et al., 2019; Booker et al., 2020; Chen et al., 2020), other health care workers (*k* = 3: Stamatiou et al., 2016; Vora et al., 2019; Vanttola et al., 2020), various types of workers (*k* = 5: Drake et al., 2004; Di Milia et al., 2013; Kalmbach et al., 2015; Kerkhof, 2018; Voinescu, 2018), industrial workers (*k* = 3: Inoue et al., 2000; Taniyama et al., 2015; Li and Fan, 2018), airline workers (Lahtinen et al., 2019), flight controllers (Mizuno et al., 2016), fire fighters (Barger et al., 2015), oil rig workers (Waage et al., 2009), and police officers (Rajaratnam et al., 2011).

The majority of studies (*k* = 20) assessed SWD using the ICSD-2 or Waage et al. (2009) measure (Drake et al., 2004; Kim et al., 2006; Waage et al., 2009; Rajaratnam et al., 2011; Flo et al., 2012; Asaoka et al., 2013; Di Milia et al., 2013; Barger et al., 2015; Taniyama et al., 2015; Anbazhagan et al., 2016; Mizuno et al., 2016; Stamatiou et al., 2016; Zaki et al., 2016; Fadeyi et al., 2018; Li and Fan, 2018; Shi and Fan, 2018; Tangkumnerd, 2018; Francy et al., 2019; Haile et al., 2019; Booker et al., 2020). In addition, seven studies used the ICSD-3 or similar criteria (Kalmbach et al., 2015; Joseph et al., 2018; Kerkhof, 2018; Voinescu, 2018; Lahtinen et al., 2019; Vora et al., 2019; Chen et al., 2020), one study used the ICSD-1 (Inoue et al., 2000), and one study (Vanttola et al., 2020) used both the ICSD-2 and ICSD-3 criteria.

The studies included a total of 22,014 participants, ranging from 26 (Lahtinen et al., 2019) to 5,771 (Barger et al., 2015) with a mean of 759.1 (*SD* = 1,356.6) participants. Of the total, 9,876 were females, whereas 881 were males (rest not accounted for in terms of sex). Table 1 presents further characteristics of the included studies.

### Prevalence Estimates and Heterogeneity

**Figure 2:**
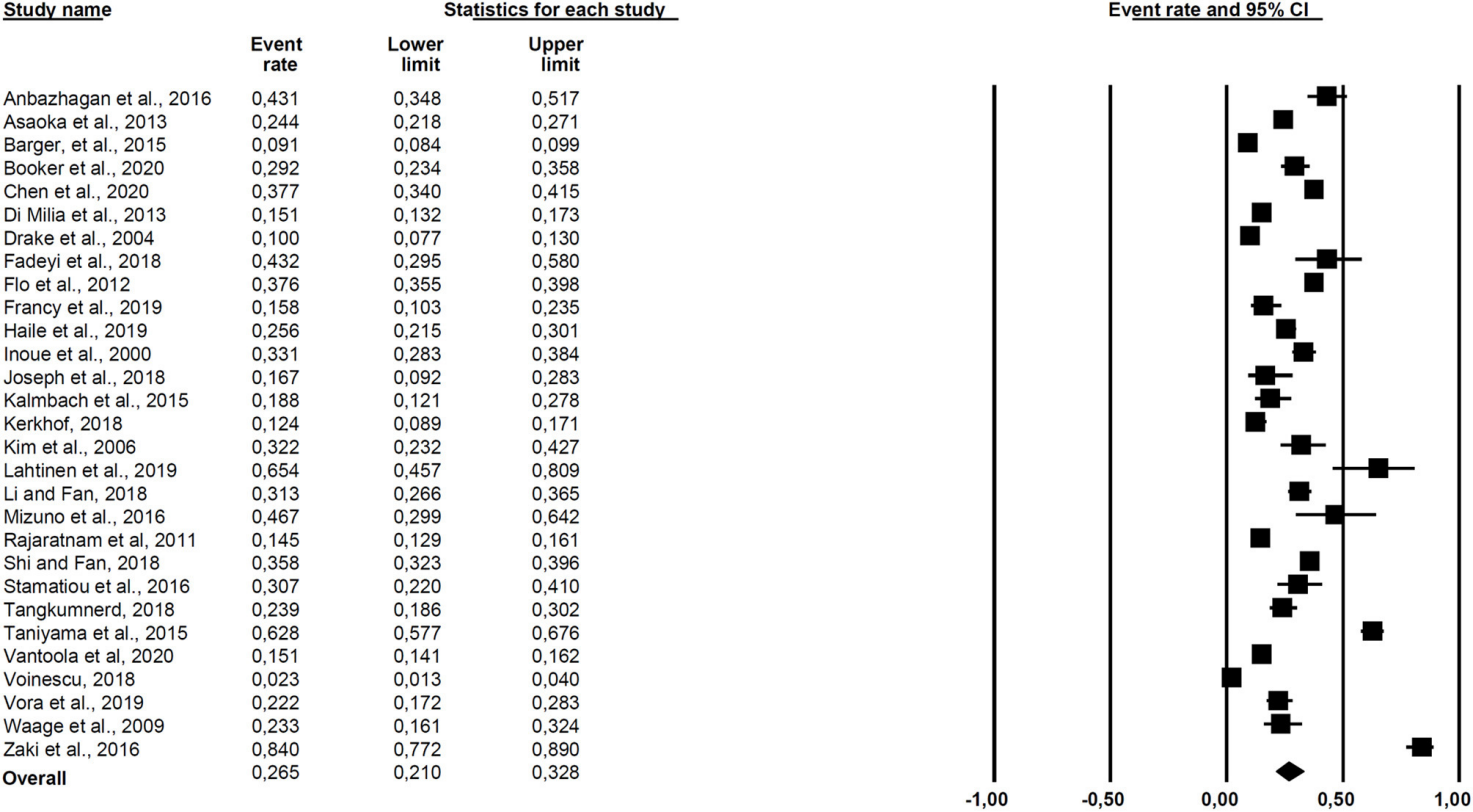
Forest plot of the included studies.

The results of the meta-analysis are presented in Figure 2. The overall prevalence across all 29 studies was 26.5% (95% CI = 21.0–32.8). Cochran *Q* was significant (*Q* = 1,845.36, *df* = 28, *p* < 0.001), suggesting heterogeneity across the prevalence estimates, and the *I*^2^ statistic was 98.5%, indicating very high heterogeneity. The 95% prediction interval was 0.06–0.67.

### Correlates of SWD Prevalence

Because of the significant heterogeneity, a metaregression analysis based on a random-effects model was conducted including diagnostic criteria (ICSD-2 = 0, ICSD-3 = 1), country of study (developed = 0, developing =1), and type of workers (not only night workers = 0, night workers only = 1) as predictors. The results are presented in Table 3. Overall, the regression model was significant (*Q* = 13.54, *df* = 4, *p* = 0.009, *R*^2^ = 33%). Diagnostic criteria (*b* = −0.914, *p* = 0.027) and the natural logarithm of sample size (*b* = −0.344, *p* = 0.006) were negatively related to SDW prevalence. Still, there was unexplained variance (*Q* = 979.51, *df* = 24, *p* < 0.001).

**Table 3:** Results of meta regression of diagnostic criteria, study country, night work and sample size on SWD prevalence.

**Predictor**	**Coefficient**	**SE**	**95% CI**	***Z***	**2-sided *p***
Intercept	1.122	0.898	−0.639 to 2.882	1.25	0.212
Diagnostic criteria	−0.914	0.418	−1.733 to −0.096	−2.19	0.027
Study country	0.415	0.322	−0.216 to 1.047	1.29	0.197
Night work	0.192	0.364	−0.906 to 2.882	−0.53	0.597
Sample size	−0.345	0.125	−0.591 to 0.099	−2.75	0.006

*Diagnostic criteria (ICSD-2 = 0, ICSD-3 = 1), study country (developed = 0, developing = 1), and night work (includes night work: no = 0, yes = 1). k = 29. R^2^ = 0*.

### Publication Bias

The results of Egger test (*b* = 3.80, 95% CI = −1.50–9.10, *t* = 1.47, *p* = 0.15) did not suggest publication bias. The trim-and-fill procedure trimmed 0 studies and consequently did not change the overall prevalence estimate.

## Discussion

A total of 29 studies fulfilled the inclusion criteria and were consequently included in the meta-analysis, amounting to an overall SWD prevalence of 26.5%. The dispersion of effect sizes was significant, ranging from 2.3% (Voinescu, 2018) to 84.0% (Zaki et al., 2016).

The prevalence of SWD was relatively high across the included studies, suggesting that approximately one in four is affected. This indicates that shift work in general takes a heavy toll on worker's health and is as such in line with several meta-analyses underlining the health detrimental effects of shift work (Stocker et al., 2014; Wang et al., 2015; Gan et al., 2018; Liu et al., 2018; Pahwa et al., 2018; Torquati et al., 2018, 2019; Garbarino et al., 2019). The finding suggests that focus on prevention and treatment of SWD should be prioritized (Gupta et al., 2019). Additionally, the overall SWD prevalence was high compared to typical prevalences estimated for insomnia in daytime workers (Drake et al., 2004; Takahashi et al., 2008; Ursin et al., 2009; Yazdi et al., 2014; Yong et al., 2017).

The high disparity of prevalences suggests that the included studies differ on several dimensions. In order to elucidate this further, a meta-regression with four independent variables was conducted. The independent variables comprised diagnostic system (ICSD-2 vs. ICSD-3), country (developed vs. developing), night work (all types of workers vs. night workers only), and sample size. The prevalences were higher when studies used the ICSD-2 diagnostic criteria (American Academy of Sleep Medicine, 2005), compared to the ICSD-3 criteria (American Academy of Sleep Medicine, 2014). This is understandable as the ICSD-3 criteria are more stringent than the ICSD-2 criteria. Sample size was also inversely related to prevalences, which might suggest the presence of a small study effect (Richter et al., 2019), although the Egger test (Egger et al., 1997) and the trim-and-fill procedure (Duval and Tweedie, 2000) suggested otherwise. Other predictors that may explain additional variance in SWD prevalence are profession (Barger et al., 2009), shift work experience (Saksvik-Lehouillier et al., 2013), codetermination of work schedule (Albertsen et al., 2008), sample characteristics such as age and sex (Saksvik et al., 2011), general working conditions (Costa, 2003), speed and direction of rotation (Knauth, 1995), shift start times (Sallinen and Hublin, 2015), and other sample characteristics such as work–family spillover (Kunst et al., 2014). Future studies should thus more stringently investigate predictors of SWD.

There was a large variation between studies in terms of study quality, and this was also strongly related to study dimension. Only two studies (Flo et al., 2012; Mizuno et al., 2016) had national representative samples of specific professions, and no study included national representative studies of workers in general. Hence, more studies on the prevalence of SWD should be based on national representative samples. Additionally, fewer than half of the studies had a sampling frame reflecting the study population or described a proper random selection of participants. Moreover, few studies used an instrument with known validity and/or reliability when assessing SWD. Hence, future studies should improve especially on these study dimensions.

In terms of assessment, both the ICSD-2 and the ICSD-3 require sleep diaries or actigraphy for fulfillment of the SWD diagnosis (American Academy of Sleep Medicine, 2005, 2014). With a few exceptions (Kim et al., 2006; Mizuno et al., 2016; Lahtinen et al., 2019), such measures were not included in the prevalence studies reviewed. This seems reasonable in the context of epidemiological research. Some validated questionnaires reflecting SWD have been developed (Barger et al., 2012), but may still be too extensive for large-scale survey studies. Hence, the need for development of a short scale validated (e.g., in terms of sensitivity and specificity) against proper diagnostic procedures would advance the field. This could also facilitate consensus in terms of operationalization of the disorder and easing comparisons across study comparisons. Another issue of which the field would benefit from reaching consensus concerns which work schedules might be relevant for the SWD diagnosis. Some scholars seem, for example, to restrict the SWD diagnosis to night workers (Barger et al., 2015; Kalmbach et al., 2015), whereas others include all types of workers, including day workers only (Flo et al., 2012; Voinescu, 2018).

Most of the included studies were cross-sectional. A few exceptions to this were noted as some studies assessed potential predictors among study participants before they started working (e.g., at nursing school) and then assessed the prevalence of SWD some time following introduction to work life (e.g., 3 and 6 months) (Chen et al., 2020). Such studies may yield other prevalences than studies conducted among well-established shift workers, due to the healthy shift worker effect (e.g., those not coping with shift work quit) (Knutsson, 2004) associated with the latter type of studies. This should also be taken into consideration when interpreting the prevalence of SWD.

### Strengths and Limitations

The present meta-analysis targeted the inclusion of gray literature, as recommended for the calculation of non-biased estimates in meta-analyses (Borenstein et al., 2009). All prevalence data and quality assessment of the included studies were coded independently by two of the authors, ensuring reliability. Searches were conducted across several databases, and no restrictions in terms of time frame were applied. The meta-analysis was conducted in line with the PRISMA guidelines (Moher et al., 2009).

Some articles presented limited study information, which made the table of study characteristics of the included studies somewhat incomplete. Still, it should be noted that the authors of the present meta-analysis contacted authors to obtain missing information. In the few cases of disagreement about study coding between the raters, agreement was reached by consulting the article in question and through discussions. However, records of initial disagreement between raters were not kept, preventing calculation of interrater reliability.

## Conclusion

The prevalence of SWD was overall high (26.5%) across the included studies, although the single estimates varied strongly. This suggests that focus on prevention and treatment of SWD should be prioritized. Diagnostic criteria (ICSD-2 = 0 vs. ICSD-3 = 1) and sample size were inversely related to SWD prevalence emphasizing the need for consensus in the field in terms of SWD assessment and sample restrictions.

## Data Availability Statement

The raw data supporting the conclusions of this article will be made available by the authors, without undue reservation.

## Author Contributions

Study conceptualization, literature search, data analysis and coding of studies were conducted by SP and DS. All authors contributed to interpretation of data, writing and revising the work critically for important intellectual content, read and approved the final version of the work to be published, and agreed to be accountable for all aspects of the work in ensuring that questions to the accuracy of any part of the work are appropriately investigated and resolved.

## Conflict of Interest

The authors declare that the research was conducted in the absence of any commercial or financial relationships that could be construed as a potential conflict of interest.
